# Short-term outcomes and mortality in older patients with breast cancer at a single tertiary center

**DOI:** 10.1016/j.sipas.2025.100313

**Published:** 2025-10-13

**Authors:** Nora Trabulsi, Nada AbuBakr Alkhateeb, Feryal Omar Attiah, Rozan Altaifi, Bana Fakeeh, Alaa Shabkah, Ali Farsi, Somayah Saeed Bawazeer, Salma Sait, Marwan Al-Hajeili

**Affiliations:** a Department of Surgery, Faculty of Medicine, King Abdulaziz University, Jeddah, Saudi Arabia; b Department of Surgery, King Abdulaziz Medical City, Jeddah, Saudi Arabia; c Department of Surgery, East Jeddah Hospital, Jeddah, Saudi Arabia; d Department of Surgery, International Medical Center, Jeddah, Saudi Arabia; e MSc of Epidemiology and Biostatistics, College of Medicine, King Abdulaziz University, Jeddah, Saudi Arabia; f Department of Medicine, Faculty of Medicine, King Abdulaziz University, Jeddah, Saudi Arabia

**Keywords:** Elderly breast cancer, Mortality, Predictors of morbidity, Short-term outcomes

## Abstract

**Background:**

Breast cancer (BC) affect women worldwide, and with a rising global incidence, it represents a burden on health systems. In Saudi Arabia, the number of cases of BC and its age distribution have notably increased. Despite this increase, data on BC characteristics, management, and outcomes in this demographic are limited.

**Methods:**

We performed this retrospective descriptive study at King Abdulaziz University Hospital in Jeddah, Saudi Arabia, spanning 2008 to 2020. It included older women (60 years or older) diagnosed with primary BC. Data from hospital records included patient demographics, comorbidities, treatments, and short-term outcomes within 30 days of treatment. We aimed to determine the significant associations of patient, disease and treatment factors with length of stay, short-term outcomes, and mortality.

**Results:**

The study included 115 older female patients with BC, with a mean age of 67 years. Comorbidities such as diabetes (39.1 %) and hypertension (40.9 %) were prevalent. Most patients were diagnosed with stage T2 (49 %) and N1 (42 %) nonmetastatic invasive ductal carcinoma (88.7 %). The recurrence rate was 21 %, while the crude all-cause mortality rate was 20 %. Short-term outcomes showed a 4.35 % readmission rate and a 2.6 % reoperation rate, with an average hospital stay of 3.61 days. Positive surgical margins, type of surgery, and the presence of metastasis significantly predicted extended hospital stays. Smoking was significantly linked to overall morbidities within 30 days.

**Conclusion:**

This study highlights the unique characteristics and treatment outcomes of older women with BC. Comorbidities, tumor stage, and receptor status are crucial for its management and outcomes. The findings emphasize the need for tailored treatment strategies, in consideration of older patients' distinct profiles. Future research should include comparative analyses with younger cohorts to establish age-specific recommendations and optimize treatment approaches for older women.

## Introduction

Female breast cancer (BC) continues to be the most common cancer diagnosed globally[1]. The global incidence of female BC cases in 2020 was >2.3 million with approximately 700,000 deaths, the fifth most common cause of cancer deaths worldwide[1]. Women aged 50 and older represented >70 % of these new cases and 81 % of the deaths,[2]. although the age distribution for incidence and mortality varies greatly between different parts of the world[2]. By 2040, it is anticipated that the annual incidence of BC cases will surge by >40 %, reaching around 3 million. In parallel, BC fatalities are expected to rise by over 50 %, from 685,000 in 2020 to approximately 1 million in 2040[2]. In North America and Europe, women who are 50 years or older represent >80 % of new cases and 90 % of mortalities; in contrast, in Middle Africa, they represent 43 % of new cases and 49 % of mortalities[1]. The burden of BC has been increasing and overwhelming global health systems, especially in this age group[1]. Over the last 30 years, there has been a notable rise in BC cases among women in the Middle East and North Africa region[3]. Research covering the years 1990 to 2019 indicates that the age-standardized rate (ASR) of BC incidence in this region reached 37.5 cases per 100,000 women in 2019. This figure marks an increase of 90.9 % since 1990[3].

In Saudi Arabia, the incidence of BC among women has shown significant trends in recent years. An earlier epidemiological study spanning 2001 to 2008 indicated that in Saudi Arabia, the most common group for BC diagnoses was women between 30 and 44 years old, accounting for 38.6 % of cases[4]. This was followed by women between 45 and 59 years old, representing 31.2 % of cases[4]. One study analyzed BC data from the Saudi Cancer Registry, showing a substantial increase in cases from 783 in 2004 to 2040 in 2016[5]. The ASR climbed from 15.4 to 27.2 per 100,000 women during this period, with a significant annual percentage change[5]. The median age at diagnosis increased from 47 to 50 years[5]. Most cases occurred in women aged 40–59 years, with the highest rise in incidence among those aged 70–74[5]. Regionally, Riyadh, Makkah, and the eastern region reported most cases, with the eastern region having the highest average ASR[5]. Significant regional increases in incidence were noted, especially in Najran, Qassim, and Hail[5].

Although the number of BC cases is increasing in older patients, data are lacking on BC characteristics, management, and survival in these age groups[6]. The management of BC in older women is considered more challenging than in younger women for many reasons, but it is primarily due to their unique health profiles[7]. Older patients often have other health conditions, limited physical mobility, dependency in daily activities, and varying cognitive functions, all of which can complicate cancer treatment. In addition, social and economic factors play a role[7].

As life expectancy increases, new approaches are required in the treatment of older patients with BC[8]. Undermanagement of these patients is not safe when it is based solely on age or hormonal manipulation, which may result in treatment resistance[8]. Older women diagnosed with BC tend to experience lower levels of sadness, worry, and stress in comparison to their younger counterparts, who may struggle more and require additional support to improve their well-being[[9], [10], [11]]. Conversely, younger patients often undergo more intensive treatment, which can negatively affect their quality of life to a greater extent than it does in older patients because of the higher likelihood of concurrent health issues[10,11]. However, the presence of this age-related difference in quality of life does not imply that older patients continuously cope better with cancer-related stress.

A study conducted in Saudi Arabia revealed that cancer is a sensitive topic, and most patients, particularly older individuals, tend to refrain from sharing and discussing their conditions[12]. In other regions, evidence suggests that older individuals with cancer experience fluctuations in their psychological, physical, and social well-being over time, including a decline in depressive symptoms, physical performance, and role effectiveness[13].

Research points out that a treatment approach that incorporates two or more therapeutic agents serves as the cornerstone of cancer therapy[14]. Specifically, studies have demonstrated that combination therapy has not only proved to be safe and well-tolerated, but it also enhances overall survival rates (6.4 months vs. 4 months) and progression-free survival when compared with currently available treatments for this particular type of cancer[15,16].

There is a gap in the literature regarding the outcomes and management patterns of BC in older women in our geographic area and country. Therefore, the objective of this study was to methodically evaluate the outcomes of BC in older women, examining both the effectiveness and the impact of various treatments on morbidity and mortality. Specifically, it focused on analyzing short-term outcomes, such as those within 30 days following the initiation of treatment and postsurgical interventions. In addition, we sought to assess overall postoperative mortality rates. The insights derived from this study are expected to enhance the understanding of BC dynamics in older women, ultimately contributing to the optimization of management and treatment strategies for this particular demographic.

## Methods

The STROCSS 2021 guidelines were followed in describing the methods used and in reporting the study findings[17].

### Study design and setting

This retrospective descriptive study was conducted at King Abdulaziz University Hospital (KAUH), a tertiary cancer center in Jeddah, Saudi Arabia, from January 1, 2008, through December 31, 2020. Ethical approval was obtained from the Research Committee at the Unit of Biomedical Ethics at KAUH (Ref number: 113–24).

### Inclusion and exclusion criteria

Older women (60 years or older) diagnosed with primary BC at KAUH between January 1, 2008, through December 31, 2020, were included in the study. Male patients, female patients younger than 60 years, and patients with benign breast conditions were excluded.

### Data collection

Patient data were meticulously extracted from the hospital's information system, encompassing a comprehensive review of histopathology and operative reports, prescribed medications, hospital admission episodes, and clinical notes. An array of variables were collected, including patient demographics, body mass index, and a spectrum of comorbidities such as diabetes, hypertension, ischemic heart disease, coronary artery bypass grafting, heart failure, peripheral vascular disease, dyslipidemia, chronic kidney disease, and smoking status.

In addition, details regarding breast surgery type, pathological and clinical staging, BC treatments, and length of hospital stay were recorded. The study also closely monitored short-term outcomes within 30 days of the initiation of treatment. These outcomes were defined as the various medical events and complications that occur within 30 days following the initiation of BC treatment, which can include neoadjuvant therapy or surgical procedures. The outcomes encompassed a range of incidents, such as hospital readmissions; reoperations; visits to the emergency room; intensive care unit (ICU) admissions; incidents of deep vein thrombosis (DVT), pulmonary embolism (PE), and cardiac events; and the overall occurrence of any morbidities. The study also included the collection and analysis of data on disease recurrence and mortality rates.

### Statistical analysis

All data were entered and analyzed by using SPSS, version 25 (IBM Corp., Armonk, NY, USA). All qualitative variables are presented as frequency and percentages. The chi-square test was applied (exact Fisher’s P value applied if necessary) to determine the significant associations in length of stay, short-term outcomes, and mortality. Statistically significant results underwent another multivariate analysis. A p value of <0.05 was considered significant.

## Results

### Patient characteristics

In this study, 115 elderly patients were included, with a mean age of 67 years (SD = 6.54). The majority were diagnosed with stage T2 (49 %) and N1 (42 %) nonmetastatic disease (88 %). Invasive ductal carcinoma (IDC) was the predominant pathological subtype (88.7 %). For the duration of this study, we observed an overall recurrence rate of 21 % and crude all-cause mortality rate of 20 %. More detailed patient characteristics are available in Table 1.

**Table 1:** Descriptive statistics of older patients with breast cancer (N = 115)a.

Characteristic	N	%
Age at diagnosis, mean, SD	67.72	6.5
Clinical T^b^		
1	25	23.1
2	53	49.1
3	16	14.8
4	14	12.9
Clinical N^b^		
0	40	37
1	46	42.6
2	20	18.5
3	2	1.8
Clinical M^b^		
0	97	88.9
1	12	11
Pathological T^b^		
0	6	5.4
1	34	30.6
2	48	43.2
3	13	11.7
4	10	9
Pathological N^b^		
0	47	41.9
1	34	30.4
2	23	20.5
3	8	7.1
Histological grade^b^		
1	21	18.9
2	56	58.6
3	25	22.5
Pathological subtype		
IDC	102	88.7
ILC	9	7.8
Other	4	3.8
ER		
Positive	79	68.7
Negative	36	31.3
PR		
Positive	72	62.6
Negative	43	37.4
HER2 status^b^		
Equivocal	4	3.5
Negative	75	65.8
Positive	35	30.7
Axillary surgery type		
SLNB	38	33
ALND	76	66
Not done	1	0.87
Breast surgery type		
BCS	43	37.4
Mastectomy	72	62.6
Bilateral breast surgery		
Yes	6	5.2
No	109	94.8
Radiotherapy		
Yes	81	70.4
No	34	29.6
Adjuvant chemotherapy		
Yes	60	52.2
No	55	47.8
Neoadjuvant chemotherapy		
Yes	27	23.5
No	88	76.5
Hormonal treatment		
Yes	75	65.22
No	40	34.8
Anti-HER treatment		
Yes	26	22.6
No	89	77.4
Margins^b^		
Negative	104	95.4
Positive	5	4.6
Lymph vascular invasion		
Negative	72	69.2
Positive	32	30.8
Recurrence		
Yes	25	21.7
No	90	78.3
Site of recurrence		
Distant	14	12.2
Locoregional and distant	5	4.3
Locoregional	6	5.2
None	90	78.3
Distant recurrence		
Yes	19	16.5
No	96	83.5
Mortality		
Yes	24	20.9
No	91	79.1
Comorbidities		
Yes	66	57.4
No	49	42.6
Hypertension		
Yes	47	40.9
No	68	59.9
Diabetes		
Yes	45	39.1
No	70	60.9
Insulin dependent		
Yes	11	9.6
No	104	90.4
IHD, CABG preoperatively		
Yes	7	6.1
No	108	93.9
Heart failure		
Yes	2	1.7
No	113	98.3
Peripheral vascular disease		
Yes	1	0.87
No	114	99.1
Stroke or TIA		
Yes	2	1.7
No	113	98.1
Dyslipidemia		
Yes	26	22.6
No	89	77.4
CKD		
Yes	4	3.8
No	111	96.5
Smoking		
Yes	4	3.8
No	111	96.5

a Data are presented as number and percentage unless otherwise indicated.

b Missing values. Abbreviations: ALND, axillary lymph node dissection; BCS, breast-conserving surgery; CABG, coronary artery bypass grafting; CKD, chronic kidney disease; ER, estrogen receptor; IDC, invasive ductal carcinoma; HER2, human epidermal growth factor receptor 2; IHD, ischemic heart disease; ILC, invasive lobular carcinoma; PR, progesterone receptor; SLNB, sentinel lymph node biopsy; TIA, transient ischemic attack.

### Short-term outcomes

Evaluation of the short-term outcomes in older patients with BC within 30 days of treatment revealed the following: a readmission rate of 4.35 %, a reoperation rate of 2.6 %, an ICU admission rate of 0.9 %, and a cardiac event occurrence of 1.7 %. The overall morbidity within 30 days was 19.1 %, as shown in Table 2.

**Table 2:** Short-term outcomes within 30 daysa.

Variable	N	%
Length of stay, mean, SD	3.6	2.9
Readmission within 30 days		
Yes	5	4.3
No	110	95.6
Reoperation within 30 days		
Yes	3	2.6
No	112	97.4
ICU stay within 30 days		
Yes	1	0.9
No	114	99.1
DVT within 30 days		
Yes	0	0
No	115	100
PE within 30 days		
Yes	0	0
No	115	100
Any cardiac event within 30 days		
Yes	2	1.7
No	113	98.1
Overall morbidities within 30 days		
Yes	22	19.1
No	93	80.9

a Data are presented as number and percentage unless otherwise indicated. Abbreviations: DVT, deep vein thrombosis; ICU, intensive care unit; PE, pulmonary embolism.

The average hospital stay for older patients with BC was 3.61 days (SD = 2.99). Univariate regression analysis identified several significant factors that influenced length of hospital stay. These factors included positive surgical margins (*p* = 0.0013), undergoing a mastectomy (*p* = 0.0002), the presence of metastatic disease (*p* = 0.032), and pathologically positive lymph nodes (*p* = 0.0005). Further multivariate analysis confirmed that positive surgical margins (*p* = 0.000), type of breast surgery (*p* = 0.019), and metastasis (*p* = 0.014) were significant predictors of extended hospital stays (*p* < 0.05). However, the influence of pathologically positive lymph nodes was not statistically significant in this analysis (*p* = 0.109). The detailed results of both univariate and multivariate analyses are presented in Table 3.

**Table 3:** Length of stay.

Characteristic	Univariate analysis (length of stay in hospital)				Multivariate analysis	
	Mean = 3.61	SD = 2.99	T test	P value	Coefficient	P value
Age			−0.76	0.45		
<65	3.4	2.2				
65+	3.8	3.5				
Margins			−3.31	0.00		
Negative	3.4	2.5			Reference	
Positive	9	9.8			8.83	0.00
Smoking			1.10	0.28		
No	3.7	3.03				
Yes	2	1.1				
Breast surgery type			−3.86	0.00		
BCS	2.2	1.8			Reference	
Mastectomy	4.4	3.3			1.37	0.02
Breast cancer type			0.11	0.95		
IDC	3.6	3.1				
ILC	3.7	2.3				
Mammary	4.5	0.7				
Other	3	3.4				
Clinical T			0.36	0.78		
1	3.3	2.4				
2	3.9	3.7				
3	3.2	2.04				
4	3.9	1.9				
Clinical N			2.03	0.11		
0	2.8	1.8				
1	4.5	4.01				
2	3.5	1.9				
3	3.5	0.7				
Clinical M			−2.17	0.03		
0	3.4	2.7			Reference	
1	5.6	4.8			2.25	0.01
ER			0.74	0.46		
Positive	3.9	3.2				
Negative	3.5	2.9				
PR			0.35	0.73		
Positive	3.7	3.1				
Negative	3.5	2.9				
HER2			0.92	0.40		
Equivocal	2.3	2.3				
Negative	3.4	2.9				
Positive	4.2	3.2				
Pathological T			1.12	0.35		
0	2.8	1.2				
1	3.04	2.1				
2	4.2	3.9				
3	3.3	1.6				
4	4.8	1.9				
Pathological N			6.49	0.00	Reference	
0	2.3	1.7			.53	
1	5	3.6				0.11
2	4.6	3.4				
3	3.85	1.1				
Adjuvant chemotherapy			0.38	0.70		
No	3.5	3.2				
Yes	3.7	2.8				
Neoadjuvant chemotherapy			−0.11	0.91		
No	3.6	2.8				
Yes	3.7	3.5				
Hormonal treatment			0.00	1.00		
No	3.6	2.15				
Yes	3.6	3.4				
Anti-HER2			−1.75	0.08		
No	3.3	2.8			Reference	
Yes	4.6	3.6			.30	0.65
Radiotherapy			0.18	0.86		
No	3.7	2.3				
Yes	3.6	3.3				
Axillary surgery type			2.72	0.07		
ALND	4.1	3.2			Reference	0.86
SLNB	2.8	2.4			.061	
Recurrence			−1.15	0.25		
No	3.4	2.6				
Yes	4.3	4.25				

Abbreviations: ALND, axillary lymph node dissection; BCS, breast-conserving surgery; ER, estrogen receptor; IDC, invasive ductal carcinoma; HER2, human epidermal growth factor receptor 2; ILC, invasive lobular carcinoma; PR, progesterone receptor; SLNB, sentinel lymph node biopsy.

Emergency department (ED) visits within 30 days were significantly associated with neoadjuvant chemotherapy, both in univariate (*p* = 0.023) and multivariate analyses (*p* = 0.041) (Table 4). Two cardiac events were reported in patients with IDC, clinical N1M0 with ER, PR, and HER2, who underwent a mastectomy. Smoking was also found to be a significant factor for overall morbidities within 30 days in the univariate analysis (*p* = 0.022) (Table 5).

**Table 4:** ED visit within 30 days.

Characteristic	Univariate analysis (ED visit within 30 days)				Multivariate analysis	
	Yes = 15	No = 100	χ^2^	P value	OR	P value
Age			1.52	0.22		
<65	9 (60 %)	43 (43 %)				
65+	6 (40 %)	57 (57 %)				
Margins			0.84	1.00*		
Negative	15 (100 %)	89 (94.7 %)		Fisher’s		
Positive	0 (0 %)	5 (5.3 %)		exact		
Smoking			4.99	0.08*		
No	13 (86.7 %)	98 (98 %)		Fisher’s	Reference	
Yes	2 (13.3 %)	2 (2 %)		exact	5.96	0.12
Breast surgery type			0.63	0.43		
BCS	7 (46.8 %)	36 (36 %)				
Mastectomy	8 (53.3 %)	64 (64 %)				
Breast cancer type IDC ILC Other	11 (73.3 %) 2 (13.3 %) 2 (13.3 %)	91 (91 %) 7 (7 %) 2 (2 %)	6.15	0.09* Fisher’s exact	Reference 2.12	0.04
Clinical T 1 2 3 4	4 (26.7 %) 6 (40 %) 1 (6.67 %) 4 (26.7 %)	21 (22.6 %) 47 (50.5 %) 15 (16.1 %) 10 (10.7 %)	3.69 0.342*	0.34* Fisher’s exact		
Clinical N 0 1 2 3	4 (26.7 %) 8 (53.3 %) 3 (20 %) 0 (0 %)	36 (38.7 %) 38 (40.9 %) 17 (18.3 %) 2 (2.15 %)	1.32 0.720*	0.72* Fisher’s exact		
Clinical M 0 1	13 (86.7 %) 2 (13.3 %)	84 (89.4 %) 10 (10.5 %)	0.10	0.67* Fisher’s exact		
ER Positive Negative	4 (26.7 %) 11 (73.3 %)	32 (32 %) 68 (68 %)	0.17	0.77* Fisher’s exact		
PR			3.77	0.05		
`Positive	9 (60 %)	34 (34 %)			Reference	
Negative	6 (40 %)	66 (66 %)			.49	0.28
HER2 Equivocal Negative Positive	0 (0 %) 11 (73.3 %) 4 (26.7 %)	4 (4.04 %) 64 (64.65 %) 31 (31.3 %)	0.85	0.87* Fisher’s exact		
Pathological T 0 1 2 3 4	1 (6.7 %) 5 (33.3 %) 7 (46.7 %) 0 (0 %) 2 (13.3 %)	5 (5.2 %) 29 (30.2 %) 41 (42.7 %) 13 (13.5 %) 8 (8.3 %)	2.53	0.57* Fisher’s exact		
Pathological N 0 1 2 3	9 (60 %) 1 (6.7 %) 4 (26.7 %) 1 (6.7 %)	38 (39.2 %) 33 (34.02 %) 19 (19.6 %) 7 (7.2 %)	4.87	0.12* Fisher’s exact		
Adjuvant chemotherapy			1.02	0.31		
No	9 (60 %)	46 (46 %)				
Yes	6 (40 %)	54 (54 %)				
Neoadjuvant chemotherapy			5.16	0.02		
No	8 (53.3 %)	80 (80 %)			Reference	
Yes	7 (46.7 %)	20 (20 %)			3.71	0.04
Hormonal treatment No Yes	4 (26.7 %) 11 (73.3 %)	36 (36 %) 64 (64 %)	0.50	0.57* Fisher’s exact		
Anti-HER2			0.07	1.00*		
No	12 (80 %)	77 (77 %)		Fisher’s		
Yes	3 (20 %)	23 (23 %)		exact		
Radiotherapy No	4 (26.7 %)	30 (30 %)	0.07	1.00* Fisher’s		
Yes	11 (73.3 %)	70 (70 %)		exact		
Axillary surgery type			1.54	0.46		
ALND	8 (53.3 %)	68 (68 %)				
SLNB	7 (46.7 %)	31 (31 %)				
Recurrence			0.25	0.74*		
No	11 (73.3 %)	79 (79 %)		Fisher’s		
Yes	4 (26 %)	21 (21 %)		exact		

Abbreviations: ALND, axillary lymph node dissection; BCS, breast-conserving surgery; ED, emergency department; ER, estrogen receptor; IDC, invasive ductal carcinoma; HER2, human epidermal growth factor receptor 2; ILC, invasive lobular carcinoma; OR, odds ratio; PR, progesterone receptor; SLNB, sentinel lymph node biopsy.

**Table 5:** Overall morbidities within 30 days.

Characteristic	Univariate analysis (overall morbidities within 30 days)			
	Yes = 22	No = 93	χ^2^	P value
Age			0.46	0.33
<65	12 (54.5 %)	40 (43.01 %)		
65+	10 (45.4 %)	53 (56.9 %)		
Margins			1.33	0.58*
Negative	22 (100 %)	82 (94.2 %)		
Positive	0 (0 %)	5 (5.6 %)		
Smoking			8.36	0.02*
No	19 (86.4 %)	92 (98.9 %)		
Yes	3 (13.6 %)	1 (1.1 %)		
Breast surgery type			0.14	0.71
BCS	9 (40.9 %)	34 (36.6 %)		
Mastectomy	13 (59.1 %)	59 (63.4 %)		
Breast cancer type			4.40	0.17*
IDC	17 (77.3 %)	85 (91.4 %)		
ILC	3 (13.6 %)	6 (6.4 %)		
Other	2 (9.09 %)	2 (2.1 %)		
Clinical T			3.06	0.43*
1	4 (19.05 %)	21 (24.1 %)		
2	10 (47.6 %)	43 (49.4 %)		
3	2 (9.5 %)	14 (16.09 %)		
4	5 (23.8 %)	9 (10.3 %)		
Clinical N			3.01	0.41*
0	5 (23.8 %)	35 (40.2 %)		
1	12 (57.1 %)	34 (39.08 %)		
2	4 (19.05 %)	16 (18.4 %)		
3	0 (0 %)	2 (2.3 %)		
Clinical M			0.06	1.00*
0	19 (90.5 %)	78 (88.6 %)		
1	2 (9.5 %)	10 (11.4 %)		
ER			0.21	0.65
Positive	6 (27.3 %)	30 (32.3 %)		
Negative	16 (72.7 %)	63 (67.7 %)		
PR			1.85	0.17
Positive	11 (50 %)	32 (34.4 %)		
Negative	11 (50 %)	61 (65.6 %)		
HER2			0.99	1.00*
Equivocal	0 (0 %)	4 (4.35 %)		
Negative	15 (68.2 %)	60 (65.2 %)		
Positive	7 (31.8 %)	28 (30.4 %)		
Pathological T			0.59	0.97*
0	1 (4.5 %)	5 (5.6 %)		
1	6 (27.3 %)	28 (31.5 %)		
2	11 (50 %)	37 (41.6 %)		
3	2 (9.1 %)	11 (12.4 %)		
4	2 (9.1 %)	8 (8.9 %)		
Pathological N			0.67	0.94*
0	9 (40.9 %)	38 (42.2 %)		
1	8 (36.4 %)	26 (28.9 %)		
2	4 (18.2 %)	19 (21.1 %)		
3	1 (4.5 %)	7 (7.8 %)		
Adjuvant chemotherapy			.52	0.47
No	9 (40.9 %)	46 (49.5 %)		
Yes	13 (59.1 %)	47 (50.5 %)		
Neoadjuvant chemotherapy			1.05	0.31
No	15 (68.2 %)	73 (78.5 %)		
Yes	7 (31.8 %)	20 (21.5 %)		
Hormonal treatment			0.68	0.41
No	6 (27.3 %)	34 (36.6 %)		
Yes	16 (72.7 %)	59 (63.4 %)		
Anti-HER2			0.34	0.56
No	16 (72.7 %)	73 (78.5 %)		
Yes	6 (27.3 %)	20 (21.5 %)		
Radiotherapy			0.61	0.43
No	5 (22.7 %)	29 (31.2 %)		
Yes	17 (77.3 %)	64 (68.8 %)		
Axillary surgery type			0.35	0.84
ALND	14 (63.6 %)	62 (66.7 %)		
SLNB	8 (36.4 %)	30 (32.3 %)		
Recurrence			0.016	0.90
No	17 (77.3 %)	73 (78.5 %)		
Yes	5 (22.7 %)	20 (21.5 %)		

Abbreviations: ALND, axillary lymph node dissection; BCS, breast-conserving surgery; ER, estrogen receptor; IDC, invasive ductal carcinoma; HER2, human epidermal growth factor receptor 2; ILC, invasive lobular carcinoma; PR, progesterone receptor; SLNB, sentinel lymph node biopsy.

### Mortality

Overall mortality was significantly correlated with several clinical and pathological factors in univariate analysis. These factors included the clinical T stage (*p* = 0.018), clinical M stage (*p* = 0.000), pathological N stage (*p* = 0.000), pathological T stage (*p* = 0.009), axillary surgery (*p* = 0.002), and recurrence (*p* = 0.000). Multivariate analysis revealed that recurrence (*p* = 0.032) and clinical M stage (*p* = 0.012) remained significantly associated with mortality. These results are detailed in Table 6.

**Table 6:** Mortality.

Characteristic	Univariate analysis				Multivariate analysis	
	Alive = 91	Dead = 24	χ^2^	P value	HR	P value
Age			0.73	0.39		
<65	43 (47.25 %)	9 (37.5 %)				
65+	48 (52.3 %)	15 (62.5 %)				
Margins			1.25	0.58*		
Negative	83 (94.3 %)	21 (100 %)		Fisher’s		
Positive	5 (5.5 %)	0 (0 %)		exact		
Smoking			0.04	1.00*		
No	88 (96.7 %)	23 (95.8 %)		Fisher’s		
Yes	3 (3.3 %)	1 (4.2 %)		exact		
Breast surgery type			1.99	0.16		
BCS	37 (40.7 %)	6 (25 %)				
Mastectomy	54 (59.3 %)	18 (75 %)				
Breast cancer type IDC	82 (90.1 %)	20 (83.3 %)	3.3	0.17* Fisher’s exact		
ILC	7 (7.7 %)	2 (8.3 %)				
Other	2 (2.2 %)	2 (8.3 %)				
Clinical T 1 2 3	22 (25.3 %) 46 (52.8 %) 12 (13.8 %)	3 (14.3 %) 7 (33.3 %) 4 (19.05 %)	10.86	0.02* Fisher’s exact		
4	7 (8.05 %)	7 (33.3 %)				
Clinical N 0 1 2 3	34 (39.1 %) 35 (40.2 %) 16 (18.4 %) 2 (2.3 %)	6 (28.6 %) 11 (52.4 %) 4 (19.05 %) 0 (0 %)	1.58	0.76* Fisher's exact		
Clinical M			13.2	0.00		
0	83 (94.3 %)	14 (66.7 %)			Reference	
1	5 (5.7 %)	7 (33.3 %)			8.90	0.12
ER			2.98	0.08		
Positive	25 (27.5 %)	11 (45.8 %)			Reference	
Negative	66 (72.5 %)	13 (54.2 %)			0.70	0.77
PR			3.65	0.056		
Positive	30 (32.97 %)	13 (54.2 %)			Reference	
Negative	61 (67.03 %)	11 (45.8 %)			0.63	0.70
HER2 Equivocal Negative Positive	3 (3.3 %) 59 (65.6 %) 28 (31.1 %)	1 (4.2 %) 16 (66.7 %) 7 (29.2 %)	0.06	1.00* Fisher’s exact		
Pathological T 0 1 2 3 4	6 (6.7 %) 27 (30.3 %) 39 (43.8 %) 13 (14.6 %) 4 (4.5 %)	0 7 (31.8 %) 9 (40.9 %) 0 6 (27.3 %)	14.9	0.01* Fisher's exact		
Pathological N 0 1 2 3	46 (51.1 %) 23 (25.6 %) 15 (16.7 %) 6 (6.7 %)	1 (4.5 %) 11 (50 %) 8 (36.4 %) 2 (9.1 %)	16.1	0.00* Fisher's exact		
Adjuvant chemotherapy			1.3	0.26		
No	46 (50.5 %)	9 (37.5 %)				
Yes	45 (49.4 %)	15 (62.5 %)				
Neoadjuvant chemotherapy			2.57	0.1		
No	72 (79.1 %)	16 (66.7 %)				
Yes	19 (20.9 %)	8 (33.3 %)				
Hormonal treatment			1.6	0.2		
No	29 (31.9 %)	11 (45.8 %)				
Yes	62 (68.1 %)	13 (54.2 %)				
Anti-HER2			0.6	0.4		
No	69 (75.8 %)	20 (83.3 %)				
Yes	22 (24.2 %)	4 (16.7 %)				
Radiotherapy			0.9	0.3		
No	25 (27.5 %)	9 (37.5 %)				
Yes	66 (72.5 %)	15 (62.5 %)				
Axillary surgery type ALND SLNB	55 (60.4 %) 36 (39.6 %)	21 (87.5 %) 2 (7.1 %)	11.5	0.002* Fisher’s exact	Reference 0.46	0.13
Recurrence			14.2	0.00		
No	78 (85.7 %)	12 (50 %)			Reference	
Yes	13 (14.3 %)	12 (50 %)			4.38	0.03

Abbreviations: ALND, axillary lymph node dissection; BCS, breast-conserving surgery; ER, estrogen receptor; HR, hazard ratio; IDC, invasive ductal carcinoma; HER2, human epidermal growth factor receptor 2; ILC, invasive lobular carcinoma; PR, progesterone receptor; SLNB, sentinel lymph node biopsy.

## Discussion

This research involved 115 older patients with BC who had a mean age of 67 years. Hypertension was the primary comorbidity in 40.9 % of cases. The predominant T-stage at diagnosis was T2, seen in 49 % of patients, and N1 in 42.6 % of patients. The majority had nonmetastatic disease.

In a study conducted at a tertiary cancer center, 390 patients were categorized into two age groups: those between 65 and 75 years and those over 75 years. The investigators reported that despite the two age groups having similar tumor characteristics, the treatment strategies differed between them[6]. Notably, women over 75 years were more likely than their younger counterparts to undergo mastectomy, but to receive less adjuvant treatment and axillary surgery[6]. This finding highlights the variability in treatment decisions based on age, even when tumor characteristics are similar[6].

A comprehensive study involving over 31,000 patients with early BC assessed the correlation between surgical treatment patterns and age, taking into account prognostic factors such as histological grade, tumor size, and lymph node involvement[18]. The findings revealed a distinct age-related trend in surgical decisions[18]. Specifically, women who were 40 years old or younger, despite exhibiting poorer histological features, had the highest rates of breast-conserving surgery (BCS)[18]. Conversely, women over 70 years showed a preference for mastectomy and were more likely to receive nonsurgical management, alongside having lower rates of BCS[18].

The research literature on short-term outcomes and comorbidities in older female patients with BC is relatively sparse. Existing studies have primarily focused on comparing the characteristics and treatment patterns of BC in older patients with those in younger cohorts.

Our study highlighted that IDC was the predominant pathological subtype of BC in older patients. This result was corroborated by findings from a study by Malik et al.,[19]. showing that 69 % of patients with BC aged 71 years and older had IDC. Similarly, in a study by Gal et al.,[6]. the authors noted that IDC was the most common subtype in 74 % of patients aged 65–75 years and 78 % in those older than 75 years. Regarding hormone receptor status, over half of the study's population had positive receptors, in keeping with the results of other studies in which 86 % of patients over 71 years were ER positive, and 46.7 % of those over 65 years had luminal A disease[19,20].

In our study, mastectomy and axillary lymph node dissection were identified as the most frequently performed surgical procedures. This finding aligns with the results of a study by Wang et al.,[18]. which noted that women over the age of 70 were significantly less likely than their younger counterparts to undergo BCS. In contrast, another study found BCS to be more common in patients aged 65 and older, particularly those between 65 and 75 years[6]. However, the likelihood of undergoing mastectomy or opting out of surgery increased in patients over 75 years[6]. These observations suggest age-related variations in surgical choices among patients with BC, older patients being more inclined to undergo a mastectomy or nonsurgical options[6].

In our study, we identified several key factors that influenced the length of hospital stay for patients with BC. These factors included surgical margins, type of breast surgery performed, and presence of clinical metastasis. In terms of ED visits within 30 days of treatment, important determinants were neoadjuvant chemotherapy and the pathological subtype of BC. In addition, smoking emerged as the sole factor to have a significant impact on overall morbidities within the same 30-day period.

Our study results revealed that recurrence and the presence of clinical metastasis had a significant role in affecting the mortality rates of patients with BC. It is important to note that the 20 % mortality rate mentioned is not breast cancer–specific mortality, but rather crude all-cause mortality. These are unadjusted rates of the study group, therefore they should be regarded cautiously in this case. A multivariate analysis conducted by Ferrigni et al[21]. that focused on women aged 80 and above with BC indicated that receiving radiation therapy and hormonal therapy, along with the clinical stage of cancer, were crucial determinants for overall survival. Another study highlighted the impact of tumor subtype on both overall survival and disease-free survival[6].

Our study findings provide valuable insights into the characteristics and short-term outcomes of older patients with BC. The high prevalence of comorbidities such as diabetes and hypertension suggests that these conditions may complicate the management of BC in this population. In addition, the majority of patients presented with stage T2 and N1 nonmetastatic IDC, highlighting the necessity for early detection and intervention.

The predominance of positive receptor status among the patients aligns with what has been reported in the literature, suggesting a favorable response to hormonal therapies. The use of mastectomy, radiotherapy, and hormonal therapy underscores the importance of a multimodal treatment approach in this demographic. The observed recurrence and mortality rates emphasize the need for continuous monitoring and personalized treatment strategies to improve long-term outcomes.

Positive surgical margins were identified as a significant predictor of extended hospital stay, likely reflecting larger tumor size and more advanced disease, necessitating more extensive surgical procedures and postoperative care. This finding underscores the importance of achieving clear margins during surgery to minimize hospital stay and associated healthcare costs.

Neoadjuvant chemotherapy was significantly associated with increased ED visits within 30 days of treatment, indicating the need for closer monitoring and supportive care for patients undergoing this treatment modality. The impact of smoking on overall morbidities within the same period highlights the importance of smoking cessation programs as part of comprehensive cancer care.

Recurrence and clinical metastasis emerged as significant predictors of mortality, reaffirming the critical role of these factors in patient prognosis. This finding underscores the necessity for aggressive management and vigilant follow-up for patients with high-risk features.

The management of breast cancer increasingly emphasizes de-escalation of surgical interventions, particularly in older women. However, a 2024 San Antonio Breast Cancer Symposium (SABCS) meta-analysis demonstrated that immediate surgery in elderly patients significantly improved disease control compared with delaying surgery until after disease progression, underscoring the potential benefit of early surgical intervention in this population[22]. In line with these findings, our data indicate that breast cancer surgery remains a safe, low-morbidity option. Meanwhile, the growing body of research aimed at minimizing interventions to optimize quality of life provides an important evidence base to guide shared decision-making and to help patients carefully weigh the risks and benefits of surgical versus non-surgical management.

This study experiences several limitations. The study's retrospective design, involving manual data collection from patient files, presents limitations such as potential data loss or inaccuracies. Given the exclusion of patients treated outside our institution, selection bias cannot be excluded. In addition, the absence of a comparative younger cohort limits the generalizability of the findings. Moreover, statistical analyses conducted on limited subgroups that include readmission, reoperation, ICU admission, cardiac events, and thromboembolic incidents should be considered exploratory and hypothesis-generating rather than conclusive. Another limitation of this study is the lack of comparison group to the younger population. However, the original hypothesis of this study was that to address that BC treatment outcomes in this age group in particular is safe and has low morbidity. Future research should incorporate a comparative approach to better understand age-specific differences in BC characteristics and outcomes, enabling more tailored and effective treatment strategies.

Overall, this study highlights the complexity of managing BC in older patients and underscores the need for individualized treatment plans that consider comorbidities, tumor characteristics, and patient preferences to optimize outcomes.

## Conclusion

This study provides a comprehensive analysis of older patients with BC, emphasizing their unique characteristics and treatment outcomes. The mean age of the patients was 67 years, with a significant portion presenting with comorbidities such as diabetes and hypertension. The majority were diagnosed with stage T2 and N1 nonmetastatic IDC. Surgical interventions were common, predominantly involving mastectomy, and radiotherapy and hormonal therapy were the main treatment modalities.

Short-term outcomes within 30 days of treatment showed low rates of readmission, reoperation, ICU admission, and cardiac events, with no cases of DVT or PE. The overall morbidity rate was 19.1 %. Hospital stay duration was influenced by factors such as positive surgical margins, type of breast surgery, and the presence of metastasis.

The study also highlights significant predictors of all-cause mortality, with recurrence and clinical metastasis playing crucial roles. Univariate and multivariate analyses identified significant factors that affected length of hospital stay, ED visits, and overall morbidities, including neoadjuvant chemotherapy and smoking.

This research underscores the necessity for tailored treatment strategies for older patients with BC, considering their distinct clinical and pathological profiles. The variability in treatment decisions based on age, despite similar tumor characteristics, calls for a more nuanced approach in managing BC in older women. The study’s findings contribute valuable insights into the short-term outcomes and influencing factors in this demographic, paving the way for improved patient care and targeted interventions. However, the study's retrospective nature and lack of a younger comparison cohort highlight the need for further research to establish more definitive conclusions and age-specific recommendations.

## CRediT authorship contribution statement

**Nora Trabulsi:** Writing – review & editing, Writing – original draft, Supervision, Methodology, Data curation. **Nada AbuBakr Alkhateeb:** Writing – original draft, Data curation. **Feryal Omar Attiah:** Data curation. **Rozan Altaifi:** Writing – original draft, Investigation. **Bana Fakeeh:** Investigation, Data curation. **Alaa Shabkah:** Writing – original draft, Methodology. **Ali Farsi:** Supervision, Methodology. **Somayah Saeed Bawazeer:** Formal analysis. **Salma Sait:** Writing – review & editing. **Marwan Al-Hajeili:** Writing – original draft, Supervision.

## Declaration of competing interest

The authors declare that they have no known competing financial interests or personal relationships that could have appeared to influence the work reported in this paper.
